# Short-Term Intravenous Sodium Nitrite Infusion Improves Cardiac and Pulmonary Hemodynamics in Heart Failure Patients

**DOI:** 10.1161/CIRCHEARTFAILURE.114.001716

**Published:** 2015-05-19

**Authors:** Julian O.M. Ormerod, Sayqa Arif, Majid Mukadam, Jonathan D.W. Evans, Roger Beadle, Bernadette O. Fernandez, Robert S. Bonser, Martin Feelisch, Melanie Madhani, Michael P. Frenneaux

**Affiliations:** From the Centre for Cardiovascular Sciences, College of Medical and Dental Sciences, University of Birmingham, Birmingham, United Kingdom (J.O.M.O., S.A., J.D.W.E., R.B., M.M.); Department of Cardiothoracic Surgery, Queen Elizabeth Hospital Birmingham NHS Trust, Birmingham, United Kingdom (M.M., R.S.B.); Clinical and Experimental Sciences, Faculty of Medicine, University of Southampton, Southampton, United Kingdom (B.O.F., M.F.); and Faculty of Medicine and Health Sciences, University of East Anglia, Norwich, United Kingdom (M.P.F.).

**Keywords:** heart failure, hemodynamics, methemoglobinemia, nitric oxide, nitrite

## Abstract

**Background—:**

Nitrite exhibits hypoxia-dependent vasodilator properties, selectively dilating capacitance vessels in healthy subjects. Unlike organic nitrates, it seems not to be subject to the development of tolerance. Currently, therapeutic options for decompensated heart failure (HF) are limited. We hypothesized that by preferentially dilating systemic capacitance and pulmonary resistance vessels although only marginally dilating resistance vessels, sodium nitrite (NaNO_2_) infusion would increase cardiac output but reduce systemic arterial blood pressure only modestly. We therefore undertook a first-in-human HF proof of concept/safety study, evaluating the hemodynamic effects of short-term NaNO_2_ infusion.

**Methods and Results—:**

Twenty-five patients with severe chronic HF were recruited. Eight received short-term (5 minutes) intravenous NaNO_2_ at 10 μg/kg/min and 17 received 50 μg/kg/min with measurement of cardiac hemodynamics. During infusion of 50 μg/kg/min, left ventricular stroke volume increased (from 43.22±21.5 to 51.84±23.6 mL; *P*=0.003), with marked falls in pulmonary vascular resistance (by 29%; *P*=0.03) and right atrial pressure (by 40%; *P*=0.007), but with only modest falls in mean arterial blood pressure (by 4 mm Hg; *P*=0.004). The increase in stroke volume correlated with the increase in estimated trans-septal gradient (=pulmonary capillary wedge pressure–right atrial pressure; *r*=0.67; *P*=0.003), suggesting relief of diastolic ventricular interaction as a contributory mechanism. Directionally similar effects were observed for the above hemodynamic parameters with 10 μg/kg/min; this was significant only for stroke volume, not for other parameters.

**Conclusions—:**

This first-in-human HF efficacy/safety study demonstrates an attractive profile during short-term systemic NaNO_2_ infusion that may be beneficial in decompensated HF and warrants further evaluation with longer infusion regimens.

Although there have been considerable advances in pharmacological and device therapies for chronic heart failure (HF) that have improved both morbidity and mortality, there has been relatively little progress in the management of decompensated HF, and the mortality of patients hospitalized with HF remains high.^1^

**Clinical Perspective on p 571**

In some patients with decompensated HF, intravenous diuretics are safe and well tolerated, but in others may be associated with a marked worsening of renal function. In these circumstances, intravenous organic nitrates are commonly used. At low doses, these agents preferentially dilate capacitance vessels but, although of lesser magnitude, also dilate resistance vessels at higher doses and reduce arterial wave reflection.^2^ They usually increase cardiac output (CO). Some patients with HF are relatively resistant to organic nitrates, and almost all patients rapidly develop tolerance during sustained infusion.^3^ An effective agent devoid of tolerance would therefore be attractive. Unfortunately, several novel pharmacological agents that have shown promise in early phase trials have not been successful in larger hard end point–driven trials.^4,5^

Sodium nitrite (NaNO_2_) has a vasodilator profile that is potentially attractive for the treatment of decompensated HF. In healthy subjects, NaNO_2_ exhibits hypoxic augmentation of its vasodilator properties when administered intravenously or intra-arterially, presumably because it is reduced under hypoxic conditions to nitric oxide (NO).^6–8^ We previously demonstrated that in healthy subjects breathing room air, intra-arterial NaNO_2_ only modestly dilated forearm resistance vessels, whereas markedly dilating forearm capacitance vessels.^6^ When these subjects breathed a hypoxic gas mixture, the forearm resistance vessel dilation was augmented.^6^ Furthermore, NaNO_2_ reduced pulmonary vascular resistance (PVR) in experimental models of pulmonary hypertension^9^ and systemic nitrite infusion substantially ameliorated the pulmonary vasoconstriction associated with systemic hypoxemia in healthy volunteers.^10^ Previous studies have suggested that nitrite therapy may not be subject to the development of tolerance.^7,11^

Based on these observations, we hypothesized that intravenous NaNO_2_ infusion may substantially increase venous capacitance and reduce PVR in patients with severe HF, leading to an increase in CO by relieving external constraint to left ventricular filling from the pericardium (pericardial constraint) and right ventricle (diastolic ventricular interaction [DVI]), whereas only modestly reducing systemic vascular resistance and therefore resulting in only a minor fall in blood pressure. We therefore undertook a proof-of-concept study to evaluate the hemodynamic effects of systemic short-term NaNO_2_ infusion in patients with severe chronic HF. We chose a brief (5 minute) infusion for safety reasons because this was a first-in-man use of intravenous nitrite in HF.

## Methods

Twenty-five patients with severe chronic HF who were undergoing right heart catheterization as part of their heart transplant assessment protocol were recruited to the study. The study conformed to the Declaration of Helsinki and was approved by the University of Birmingham (ERN10-0247) and Local Research Ethics Committee (08/H1207/67). Each patient gave written informed consent after satisfying the inclusion criteria (aged ≥18 years, admission to hospital for pulmonary artery catheterization, under the transplant protocol) and exclusion criteria (preexisting inotrope therapy, recent (3 months) myocardial infarction, G6PD deficiency, and women of child-bearing potential or nursing mothers). The patients had a diagnosis of HF with reduced left ventricular (LV) ejection fraction (<40%) of various etiologies, and all patients were on optimal tolerated standard HF therapy (Table 1). Baseline characteristics of the patients enrolled in the study are listed in Table 1.

**Table 1.**
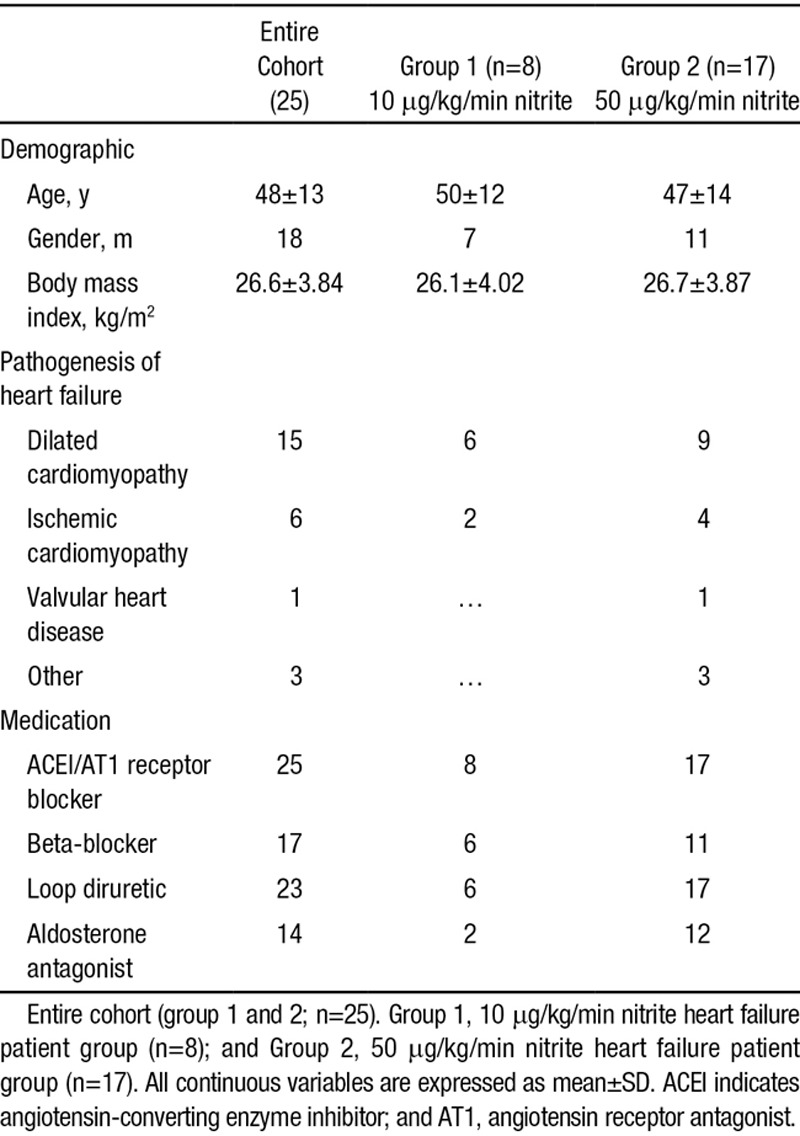
Demographics and Cardiovascular Disease Profile of the Heart Failure Patients

### Right Heart Catheterization

Patients were randomized (on a 1:2 basis; Table 2) to receive either 10 μg/kg/min (group 1; n=8) or 50 μg/kg/min (group 2; n=17) of NaNO_2_ (Martindale Pharmaceuticals, UK). The patients were then placed in a supine position to allow the insertion of a Swanz–Ganz catheter via a sheath into the right internal jugular vein under local anesthesia with positioning confirmed by fluoroscopy and pressure waveform. After 15 minutes of stabilization at baseline, the following pressures were measured at end-expiration: pulmonary arterial pressure, pulmonary capillary wedge pressure (PCWP), and right atrial pressure (RAP). The trans-septal pressure gradient (TSG) was estimated as PCWP-RAP. CO was measured using the Fick technique from the mixed venous and arterial oxygen saturations and from predicted oxygen consumption. Systemic vascular resistance (Wood units) was calculated as (mean arterial pressure–RAP/CO). Arterial elastance (a lumped measure of pulsatile and static LV afterload) was calculated as (0.9×systolic arterial pressure/stroke volume [SV]).^12^ Left ventricular stroke work was calculated as SV×mean arterial blood pressure (MABP). Arterial oxygen saturation was measured by pulse oximetry. The measurements were repeated 5 minutes later to ensure stability. NaNO_2_ was then infused intravenously over 5 minutes, and the above hemodynamic measurements were repeated at the end of the infusion. Blood pressure, ECG, and oxygen saturation were monitored continuously.

**Table 2.**
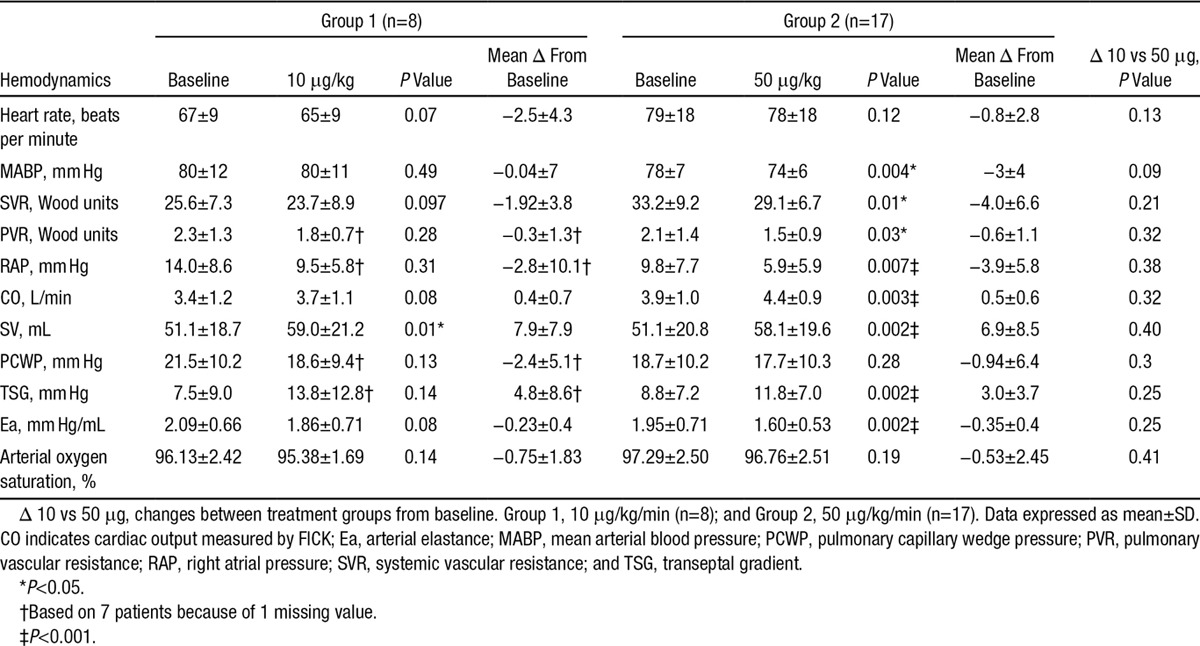
The Effect of Short-Term Sodium Nitrite Infusion on Cardiac and Pulmonary Hemodynamics in Heart Failure Patients

### Blood Samples

Venous blood samples were taken at baseline and after infusion of NaNO_2_ for methemoglobin, plasma nitrite/nitrate, and total nitroso species (RXNO) measurements. Blood for determination of venous plasma nitrite, nitrate, and RXNO concentrations were collected into EDTA tubes and immediately centrifuged (800*g* for 10 minutes at 4°C). Samples were stored at −80°C before assay. Plasma nitrite, nitrate, and RXNO concentrations were determined, after addition of *N*-ethylmaleimide (10 mmol/L final concentrations) during the thawing process, via high-performance liquid chromatography and chemiluminescence as previously described.^13^

### Statistical Analysis

Data are presented as mean±SD. Hemodynamic comparisons were performed by paired 1-tailed Student *t* test. Changes from baseline between treatment groups were performed by unpaired 1-tailed Student *t* test; a *P* value of <0.05 was taken to indicate statistical significance. Changes in TSG (ie, left ventricular end-diastolic pressure-right ventricular end-diastolic pressure) during NaNO_2_ infusion were estimated as change in PCWP–RAP.^14^ Statistical analysis was undertaken using Prism software (version 5.0, GraphPad Software, CA).

## Results

### Demographics

The demographics for the entire cohort and the groups receiving 10 μg/kg/min (group 1) and 50 μg/kg/min (group 2) are shown in Table 1. Hemodynamic parameters are shown in Table 2. At baseline (ie, before infusion of sodium nitrite), hemodynamic parameters were similar in group 1 and group 2, except heart rate was significantly higher in group 2 (*P*=0.046) and systemic vascular resistance (SVR) was significantly higher in group 2 (*P*=0.03).

### Blood Pressure and Heart Rate Effects

As shown in Table 2, there was no statistically significant change in heart rate at either infusion rate. 10 μg/kg/min NaNO_2_ infusion did not significantly affect MABP, whereas in contrast 50 μg/kg/min of NaNO_2_ infusion modestly but significantly decreased MABP by a mean of 4 mm Hg (*P*<0.004).

### Nitrite Infusion Decreases Pulmonary and Systemic Vascular Resistance

As shown in Table 2, 50 μg/kg/min of NaNO_2_ infusion significantly decreased PVR by 29% (*P*=0.03), and systemic vascular resistance fell by 12% (*P*=0.01; Table 2). Arterial elastance fell by 18% from 1.95±0.71 to 1.60±0.53 mm Hg/mL (*P*=0.002; Table 2). Infusion of 10 μg/kg/min NaNO_2_ infusion resulted in directionally similar effects, but these were not significant.

### Nitrite Reduces Pulmonary Capillary Wedge Pressure and Right Atrial Pressure and Improves Cardiac Output

As shown in Table 2, in the 50 μg/kg/min group, there was a significant reduction in mean RAP by 40% and PCWP fell by 7% (nonsignificant). Consequently, estimated TSG significantly increased by a mean of 3 mm Hg. CO significantly increased by 13% and SV significantly increased by 14%. 10 μg/kg/min NaNO_2_ infusion resulted in a significant increase in SV by 15.5%, but the increase in CO was not significant. As shown in the Figure, the change in SV during 50 μg/kg/min nitrite infusion was significantly correlated with the change in estimated TSG (*r*=0.67; *P*=0.003). Mean arterial oxygen saturation remained unchanged at either infusion rate.

**Figure.**
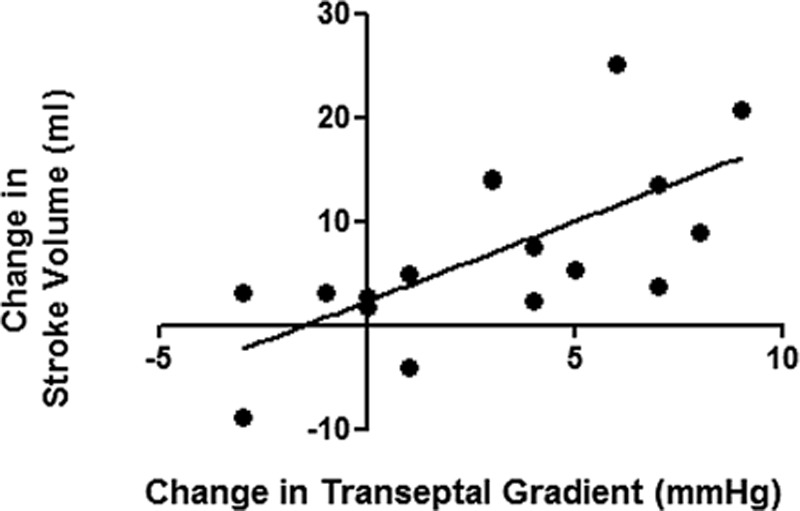
Changes in estimated trans-septal gradient positively correlated with change in stroke volume (SV) in all patients infused with 50 μg/kg/min sodium nitrite.

### Impact of Baseline SVR on SV Response to IV Nitrite

Patients receiving the higher dose infusion regime were divided into those with SVR above versus below the mean for the group at baseline (33.17 wood units). There was no significant difference in the change in LV SV between these 2 groups (9.9+8.5 versus 5.1+8.8 mL; *P*=0.3).

### Changes in SV in Patients With PCWP > and <15 mm Hg

In 11 patients with PCWP >15 mm Hg (mean 22.4±8.0 mm Hg), infusion of sodium nitrite at the higher concentration increased SV by 20% from 43.22±21.5 to 51.84±23.6 mL (*P*=0.003), whereas in those with PCWP <15 mm Hg (n=6), there was no significant change in SV (62.5±22.02 to 65.1±21.09 mL; *P*=0.24). Estimated TSG increased by 3.6 mm Hg in the high PCWP subgroup (from 11.9±6.8 to 15.5±4.7 mm Hg; *P*=0.005) and by 2 mm Hg in the lower PCWP subgroup (from 3.2±3.4 to 5.2±5.3; *P*=0.13).

### Short-Term Intravenous Nitrite Infusion Increases Circulating NO Metabolites

As shown in Table 3, 10 μg/kg/min NaNO_2_ infusion did not significantly alter plasma RXNO or plasma nitrate concentrations but significantly increased plasma nitrite. 50 μg/kg/min NaNO_2_ infusion significantly increased all measured NO metabolites from baseline.

**Table 3.**
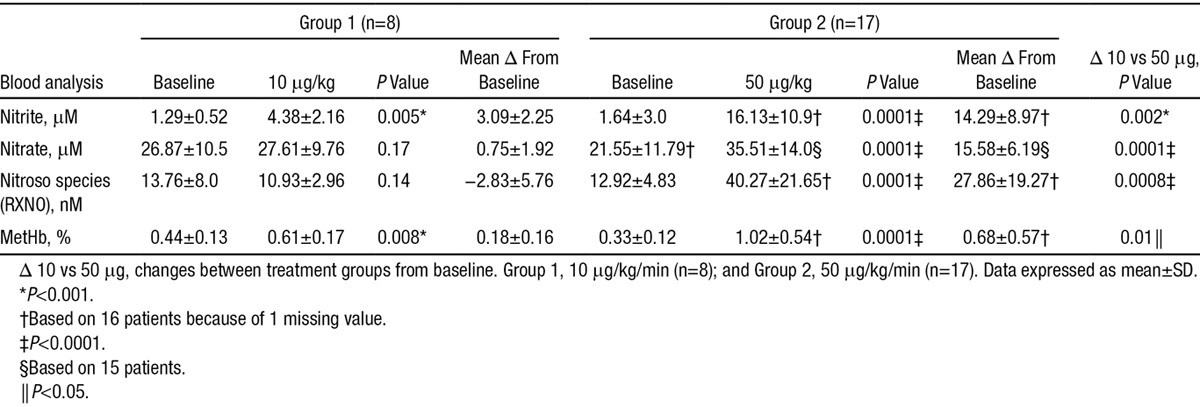
Plasma Concentrations of Nitrite, Nitrate, and Total Nitroso Species (RXNO) and Methemoglobin (MetHb) Content of Venous Blood of Heart Failure Patients at Baseline and Post Sodium Nitrite Infusion

### Methemoglobinemia Increased Within Safe Levels

As shown in Table 3, a modest dose-dependent increase in methemoglobin was observed after 10 and 50 μg/kg/min of sodium nitrite.

## Discussion

Herein, we demonstrate for the first time the short-term hemodynamic effects of intravenous NaNO_2_ in patients with severe but stable chronic HF. As hypothesized, effects were favourable, with an increase in LV SV and CO and with only a minor reduction in blood pressure. As anticipated, there was a substantial reduction in PVR, but only a modest reduction in SVR. The hemodynamic effects were statistically significant at 50 μg/kg/min. At 10 μg/kg/min, directionally similar effects were observed, but apart from a significant increase in SV, these were nonsignificant. There was no significant difference in the magnitude of the hemodynamic effects seen with 10 versus 50 μg/kg/min, but given the smaller sample size of the former group, caution should be exercised in drawing conclusions about the dose-response relationship.

The patients studied had severe but stable chronic HF and were undergoing assessment as candidates for cardiac transplantation. We chose a brief (5 minute) infusion regime as a proof of concept for safety reasons because this was a first-in-man study of systemic nitrite infusion in chronic HF. The effects were observed at the 50 μg/kg/min infusion rate and, although directionally similar, were not significant at the 10 μg/kg/min infusion rate, although the latter may partly be because of a smaller sample size in the lower dose group. Although only modest increases in methemoglobin levels were observed with either dose, based on studies in healthy volunteers, sustained infusion of 50 μg/kg/min would be expected to cause marked (and potentially dangerous) methemoglobinemia. However, we have subsequently undertaken pilot studies (data not shown) in patients with chronic HF (without hemodynamic assessment) and have demonstrated that sustained infusion of 10 μg/kg/min caused a progressive increase in plasma nitrite and RXNO, such that by 3 hours these levels were almost as high as we observed in the present study with 50 μg/kg/min infused over 5 minutes, without inducing dangerous methemoglobinemia and without inducing significant hypotension. In a study in healthy volunteers, Pluta and colleagues reported that sustained infusion (several hours) of doses of ≈5 μg/kg/min sodium nitrite resulted in significant hypotension and methemoglobin >5%.^15^ Further studies are therefore warranted to evaluate whether sustained infusion of lower doses of NaNO_2_ would have similar favorable hemodynamic effects to those observed with short-term infusion of 50 μg/kg/min without inducing methemoglobinemia or resulting in hypotension. Although patients with decompensated HF but without frank pulmonary edema and associated arterial hypoxemia may be expected to exhibit preferential dilation of capacitance vessels and pulmonary vasculature, in the latter setting, substantial resistance vessel dilation might be anticipated with an attendant fall in blood pressure.^6^ Preferential vasodilation of pulmonary vessels to underventilated alveoli has the potential to worsen ventilation–perfusion matching; however, we observed no reduction in arterial oxygen saturations in this study, despite a substantial fall in PVR.

The increase in SV in the face of a reduced PCWP represents an apparent descending limb of the Starling curve. This is because in severe chronic HF, despite high left ventricular end diastolic pressures, LV filling is impeded by external constraint from the stretched pericardium (pericardial constraint) and via the interventricular septum from the right ventricle (DVI),^16^ usually in the context of pulmonary hypertension. By preferentially dilating the systemic capacitance and pulmonary resistance vessels,^10^ nitrite may be expected to reduce RV volume and hence pericardial stretch, thereby augmenting LV filling and SV. Consistent with relief of DVI as an important mechanism, the increase in SV was only observed in the group of patients with PCWP >15 mm Hg—a cutoff that we have previously shown to identify HF patients with significant DVI.^16^ In this subgroup, the SV increased by 20%.^16^ In accordance with this concept, the reduction in RA pressure (an indirect measure of both RVEDP and pericardial pressure)^17^ was greater than the reduction in PCWP; hence, the estimated TSG (ie, the pressure gradient across the interventricular septum at end diastole)—a measure of the true filling pressure of the LV at end diastole^18^—was increased by sodium nitrite. Furthermore, the increase in SV was significantly correlated with the change in estimated TSG. We cannot exclude a significant direct myocardial effect of nitrite as a contributory mechanism. In the vertebrate, heart nitrite positively modulated the Frank–Starling response via a NO-dependent mechanism.^19^ In contrast, another study reported negative inotropic effects via a NO/cGMP-dependent mechanism in the Langendorff rat heart.^20^ In a recent study, chronic oral inorganic nitrite supplementation ameliorated the development of HF in a murine thoracic aortic constriction model in association with an upregulation of cytoprotective pathways.^21^

Intravenous sodium nitroprusside is sometimes used in the treatment of acute decompensated HF. Fifer et al reported the effects of intravenous sodium nitroprusside in patients with severe CHF.^22^ Cardiac index increased substantially (by 25%), but systemic vascular resistance also fell substantially (by 25%) and MABP fell by 13 mm Hg. The reduction in mean PCWP (by 14 mm Hg) was substantially greater than that of RA pressure (by 4 mm Hg), that is, mean TSG fell substantially, which suggests that relief of DVI was not an important mechanism of the increase in CO.^22^

Organic nitrates are more commonly used in the treatment of decompensated HF. Indeed intravenous isosorbide dinitrate has been shown to be superior to either intravenous furosemide^23^ or positive airways pressure^24^ in the management of patients with acute pulmonary edema. These agents dilate capacitance and resistance vessels.^25^ At lower doses, the vasodilator effects of glyceryl trinitrate (GTN) are predominantly on capacitance vessels, but at higher doses, effects on vascular resistance are increasingly observed.^26^ Rabinowitz et al reported the hemodynamic effects of intravenous isosorbide dinitrate in patients with decompensated HF. CO increased similarly to our study (by 17%), but the reduction in systemic vascular resistance (35%) and the fall in mean arterial pressure (10 mm Hg) were substantially more than we observed with sodium nitrite. The increase in CO was substantially greater in those patients with high resting SVR, suggesting that afterload reduction may have been an important contributor to the increase in CO.^27^ Armstrong et al reported the effects of GTN infusion in patients with severe CHF. CO increased by ≈20%. Systemic vascular resistance fell by ≈21% and MABP by 7 mm Hg. In contrast to our findings with nitrite, the fall in PCWP (by 8 mm Hg) was greater than that of RAP (by 5 mm Hg), indicating that overall the estimated TSG fell with this therapy rather than the increase we observed with sodium nitrite infusion.^28^ However, Dupuis and colleagues showed that during sustained (72 hour) infusion of GTN, SV increased in a subgroup of patients in whom LV end diastolic volume increased and fell in those in whom LV end diastolic volume fell during GTN infusion.^29^ These data suggest that GTN may relieve DVI in some patients with decompensated HF, but this effect seems less marked than we have observed in this study with intravenous sodium nitrite.

The reduction in SVR (by 12%) observed in the present study was substantially less than that observed in the above studies with either sodium nitroprusside (25%)^22^ or isosorbide dinitrate (35%),^27^ suggesting that this may play a less important role in the increase in CO with sodium nitroprusside. Furthermore, in our study, there was no significant difference in the change in SV induced by sodium nitrite between those with higher versus lower systemic vascular resistance before infusion. However, left ventricular afterload has a pulsatile component, as well as a static component, and changes in SVR do not therefore completely describe effects on LV afterload. GTN has previously been shown to reduce wave reflection.^2^ In the present study, the reduction in arterial elastance (a measure of LV afterload encompassing both static and pulsatile components) was 18%.

In summary, our data are consistent with an effect of sodium nitrite on SV largely mediated via relief of DVI because of relatively selective and potent dilation of capacitance vessels and pulmonary vasculature.^6,10^ Based on changes in estimated TSG, this mechanism may be less marked with organic nitrates and sodium nitroprusside, and changes in LV afterload may be relatively more important for these drugs than with sodium nitrite.

Nitrite has further characteristics that may make it a potentially attractive agent for the treatment of decompensated HF and therefore worthy of further investigation based on the findings of this short-term proof of concept/safety study. Some patients with HF exhibit nitrate (and NO) resistance, potentially because of increased oxidative stress.^30–32^ In contrast, during intra-arterial infusion of NaNO_2_, we observed an enhanced response in patients with HF versus controls.^11^ Furthermore, organic nitrate therapy is subject to the rapid development of tolerance. In primates, tolerance was not observed with sodium nitrite.^7^

### Study Limitations

Although nitrite infusion resulted in clear increases in plasma nitrite concentrations at both infusion rates, only the one associated with a concomitant elevation in circulating RXNO levels increased CO. This suggests that, in this setting, the beneficial hemodynamic effects of nitrite are associated either with the involvement of a post-translational modification of cardiac tissue proteins^33^ or some form of NO delivery from a circulating plasma storage form of NO (perhaps nitrosated albumin)^13^ to heart and vasculature. Although intriguing, establishing the mechanistic basis for this observation was well beyond the scope of the present study.

### Clinical Implications: Future Studies

This is the first-in-man proof of concept/safety study demonstrating a potentially favorable hemodynamic response to short-term NaNO_2_ infusion in patients with severe chronic HF. Further studies are warranted to assess longer term safety and hemodynamic efficacy, and if these are confirmed, this may warrant a randomized controlled trial of sodium nitrite versus current therapy in decompensated HF focussing on hard end points. Sodium nitrite could be administered either intravenously in such studies or perhaps in nebulized form,^34^ the latter may be particularly attractive in decompensated HF. Unfortunately, several therapies shown to have attractive acute hemodynamic profiles have failed in phase 3 trials in decompensated HF.^35^ However, a notable exception was a recent study of Serelaxin in patients with acute HF with dyspnea, in which a significant reduction in dyspnea score and 6 month mortality was reported.^36^

## Acknowledgments

We thank Dr Peter Nightingale for his statistical guidance.

## Sources of Funding

This work was supported by the UK Medical Research Council [grant number G1001536].

## Disclosures

None.

## CLINICAL PERSPECTIVE

Nitrite exhibits hypoxia dependent vasodilator properties. We therefore hypothesized that by preferential dilation of venous capacitance vessels and pulmonary arterioles (low ambient oxygen) with lesser effects on systemic arterioles (higher ambient oxygen tension), sodium nitrite would increase stroke volume in patients with heart failure by relief of pericardial constraint and diastolic ventricular interaction. We therefore performed a short-term (5 minute) proof of concept/safety study in 25 patients with severe but stable chronic heart failure who were undergoing precardiac transplant assessment. As hypothesized, there was a marked reduction in pulmonary vascular resistance and right atrial pressure with a lesser fall in pulmonary capillary wedge pressure and a modest reduction in systemic vascular resistance. Estimated trans-septal gradient (ie, pulmonary capillary wedge pressure– right atrial pressure) therefore increased, and this was correlated with the observed increase in stroke volume consistent with relief of diastolic ventricular interaction as an important mechanism. This is a potentially attractive hemodynamic profile in decompensated heart failure, but further studies are required to assess the safety and efficacy of longer-term infusion.
